# Analysis, evaluation and adaptation of the ReTransQoL: a specific quality of life questionnaire for renal transplant recipients

**DOI:** 10.1186/1477-7525-11-148

**Published:** 2013-08-30

**Authors:** Davy Beauger, Stéphanie Gentile, Elisabeth Jouve, Bertrand Dussol, Christian Jacquelinet, Serge Briançon

**Affiliations:** 1 Faculté de Médecine, Laboratoire de Santé Publique, Université Aix-Marseille, Marseille EA 3279, France; 2 CHU Marseille, Service Santé Publique et Information Médicale, Marseille, France; 3 CHU Marseille, Centre de Néphrologie et de Transplantation Rénale, Marseille, France; 4 Agence de la Biomédecine, Paris, France; 5 CHU Nancy, Epidémiologie et Evaluation Cliniques, Nancy, France; 6 Université de Lorraine, Université Paris Descartes, Apemac, Nancy EA 4360, France

**Keywords:** Quality of life, Medical outcomes, Specific questionnaire, Renal transplantation, RETRANSQOL

## Abstract

**Background:**

End stage renal disease (ESRD) profoundly impacts the lives of patients. Kidney transplantation provides the greatest health-related quality of life (HRQOL) improvement. Its measurement has become an important outcome parameter and a very important criterion in the evaluation of any type of medical treatment, especially in the field of renal transplantation.

In 2007, a specific self-administered questionnaire for renal transplant recipients was developed in the French language: the ReTransQol (RTQ).

After 5 years of use, the properties of the RTQ needed to be re-evaluated in a larger sample.

This paper describes the analysis of the ReTransQol and its adaptation to achieve an improved and revised version.

**Methods:**

The study design included three analysis phases for two samples of adult renal transplant recipients which came from two cross-sectional multicenter studies carried out in France in 2007 and 2012. Psychometrics properties like construct validity, acceptability and feasibility, reliability and convergent validity were evaluated and every analysis resulted in a new version of the questionnaire: the RTQ V2. The construct validity of the new RTQ was assessed with a Confirmatory Factor Analysis on a large sample of patients.

**Results:**

The study samples included 1,059 patients and 1,591 patients, respectively.

After a principal component analysis, item reduction was performed and a total of 13 items were deleted. A final version of the RTQ V2 was created and comprised of 32 items describing 5 domains: Physical Health, Social Functioning, Medical Care, Treatment and Fear of Losing Graft.

The explained variance between the first and second RTQ versions improved from 46.3% to 53.1%. All psychometric properties of RTQ V2 were satisfactory: IIC >0.4, IDV (%) of 100% and Cronbach’s Alpha >0.7 in every dimension. The confirmatory analysis showed that the overall scalability of the RTQ V2 was satisfactory; all items showed a good fit to the Rasch model within each dimension, and showed INFIT statistics inside the acceptable range.

**Conclusions:**

Psychometric properties allow this new version of the questionnaire to be used to assess different specific dimensions for the renal transplant population, more effectively than previously possible.

## Introduction

End stage renal disease (ESRD) profoundly impacts the lives of patients, leading to activity limitations, social participation restrictions, and dependence caused by the need for renal replacement therapy (RRT) [1-5]. It also represents a significant burden to society caused by the high treatment costs and an increasing prevalence of ESRD [6-9].

The number of patients being treated for ESRD globally was estimated to be 2,786,000 at the end of 2011, and with a 6-7% growth rate, continues to increase at a significantly higher rate than the world population. Of these ESRD patients, approximately 2,164,000 were undergoing dialysis treatment and around 622,000 were living with kidney transplants [10].

In France, between 9,000 and 10,000 new patients with chronic renal failure required the initiation of RRT in 2010, and about 67,000 were treated for renal failure, including approximately 30,000 transplants [11].

As ESRD reduces life expectancy, renal transplantation has become worldwide the treatment of choice over hemodialysis, which remains the only treatment for the majority of patients [12-14]. The aim of renal transplantation is not only to improve renal function, but also to enhance the patient’s ability to enjoy life as fully as possible. Kidney transplantation also provides the greatest improvement for health-related quality of life (HRQOL), whose measurement has become an important outcome parameter [15-17]. HRQOL has become a very important criterion in the evaluation of any type of medical treatment [18], especially in the field of renal transplantation, and is associated with the improvement of graft survival [19-22]. Several determinations of HRQOL focus on physical status and symptoms, functional status, mental health, social functioning, and general health perceptions.

Currently, few specific HRQOL questionnaires have been developed for renal transplant recipients and few are validated or available in French [23,24]. Among questionnaires adapted to the general population, the Short Form-36 Health Survey (SF-36) remains the most widely used in quality of life studies [25-27].

In 2007, a specific self-administered questionnaire for renal transplant recipients (RTR) was developed by Gentile et al. in the French language: the ReTransQol (RTQ) [28]. The RTQ was validated in a study of 335 French patients randomly selected from the registry of the transplant center in Marseille.

After 5 years of use in many French studies [29,30], the properties of the RTQ needed to be re-evaluated in a larger sample. In fact, the RTQ has been validated but not established with a Confirmatory Factor Analysis, and the psychometric properties can be improved. Moreover, the questionnaire seems to be slightly lengthy, and can be better adapted for routine use.

This paper describes the analysis of the ReTransQol and its adaptation, in order to achieve an improved and revised version.

## Methods

**Figure 1:**
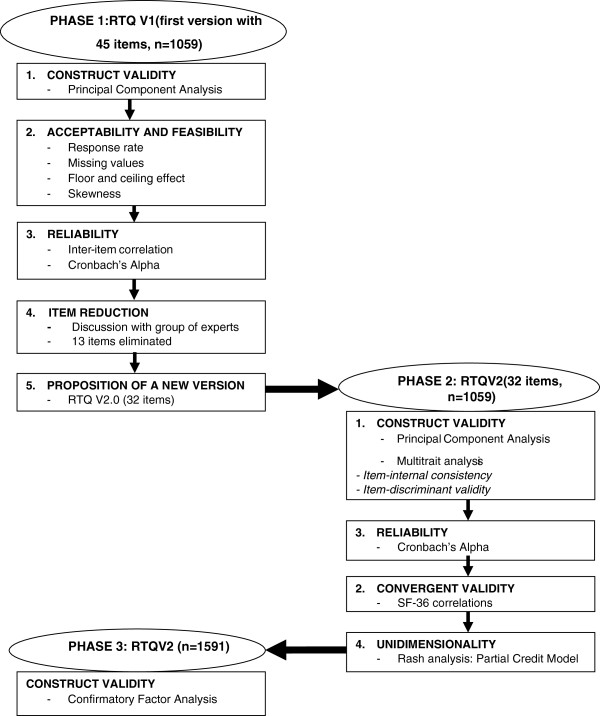
Study design: analysis and adaptation of the ReTransQoL questionnaire.

The study design involved analysis of the original version of the ReTransQol (RTQ V1) and its adaptation, and included three phases for two samples of renal transplant recipients (Figure 1).

### Data sources

For Phase 1 and Phase 2, both samples came from the same cross-sectional multicenter study, which was carried out in France between March 2007 and March 2008 in eight regions of France participating in the Renal Epidemiology and Information Network (REIN) in 2003.

For Phase 3, the sample came from a cross-sectional multicenter study (QUAVIREIN: French translation of Renal Quality of Life study) which was carried out in France between May 2011 and September 2012, in 21 regions of France participating in the REIN network in 2010 and whose register was complete and exhaustive.

### Participants

For each sample, all participants were RTR over 18 years of age with a functioning graft for at least one year. Multi-organ transplant patients before or simultaneously with their kidney transplant were excluded. The samples were stratified by regions and age class, using the same sampling rate for each stratum. The sample size was calculated to detect a difference of 5 points in the SF-36, considering a standard deviation of 20, and assuming a two-sided level of 5% with 80% power. The sample size calculation was 1,000 patients for the first national study, and 1,500 for the QUAVIREIN study. Considering a non-response rate of approximately 30% for each study, 1,300 and 1,800 questionnaires were sent in order to achieve these objectives. The number of self-administered questionnaires returned was 1,059 for the first study and 1,591 for the second, with a response rate of over 70% for both.

### Data collection procedures for both studies

The French version of the SF-36 [31] is a generic instrument, with scores ranging from 0 to 100 (worst to best possible HRQOL) for eight domains: physical functioning (PF), role-physical (RP), bodily pain (BP), general health (GH), vitality (VT), social functioning (SF), role-emotional (RE) and mental health (MH).

The ReTransQol V1 [28] is a specific instrument consisting of 45 items describing 5 dimensions: physical health (PH), mental health (MH), medical care and satisfaction (MC), treatment (TR) and fear of losing the graft (FLG). Each score ranges from 0 to 100 and the higher the score, the better the perceived state of health. Data collection included demographic, socio-demographic and medical characteristics, as well as HRQOL. SF-36 and RTQ V1 (first version) were used to evaluate HRQOL for both studies. All measuring instruments were sent to the patient’s home with a newsletter and an explanatory letter signed by the project coordinator. Patients returned the completed questionnaires via a pre-stamped envelope. Non-respondents were reminded by a second letter three weeks later and contacted by phone at the same time.

### Ethical aspects

The studies’ methodology was approved by the local Institutional Review Board, the Advisory Committee on Information Processing in Health Research (CCTIRS n°06-311 and no. 05–142) and the French consulting committee for treatment of information in health research (CNIL n°906248 and CNIL n° 905263), which ensures the confidentiality of all information collected.

### Statistical analysis

During the first phase, the construct validity and dimensional structure of the questionnaire were assessed by studying principal component factor analyses with Varimax rotation. Prior to the extraction of the factors, several tests were used to assess the suitability of the respondent data for factor analysis. These tests included Kaiser-Meyer-Olkin (KMO) Measure of Sampling Adequacy and Bartlett’s Test of Sphericity. The KMO index ranges from 0 to 1, with 0.50 considered to be suitable for factor analysis [32]. The Bartlett’s Test of Sphericity should be significant (p<0.05) for factor analysis to be appropriate. Next, a Principal Component Analysis (PCA) of the RTQ V1 was assessed to analyze the structure of the questionnaire over a larger sample than during the phase of development and validation [33]. Each dimension was examined in order to indicate which items could potentially be deleted due to their low psychometric performance at a dimension level. This was done by studying the inter-item and inter-dimension correlations (Pearson’s *r*). Items which loaded <0.40 for all the factors were considered as potentially removable.

Acceptability and feasibility were assessed regarding response rate, missing values, skewness, inter-item correlation and floor or ceiling effects. Items were eliminated during the reduction phase with missing values exceeding 5%, high inter-item correlation (r >0.80), floor or ceiling effects over 70%, or an absolute value of skewness exceeding 4.0.

During the item reduction phase, the most clinically relevant items were kept and lowest items were eliminated after analysis and discussion with the group of experts.

This analysis resulted in a new version of the questionnaire: the RTQ V2.0.

During the second phase, the psychometric properties of the RTQ V2.0 were evaluated.

Another factor analysis (PCA) with the items retained was conducted to ensure the construct validity of the RTQ V2.0. The relevance of the grouping of items into the original structure isolated by PCA was examined using multitrait multi-item analyses [34], examining correlations between item scores and dimension scores. Item-internal consistency (IIC) was assessed by correlating each item with its dimension. Item-discriminant validity (IDV) was assessed by determining the extent to which items correlate more highly with dimensions they are hypothesized to represent than with different dimensions. Each item should be highly correlated with its scale, thus supporting item-internal consistency (IIC); a correlation corrected for overlap of at least 0.4 is recommended [35]. In addition, items should be more highly correlated with their own scale than with the other dimension scales (item-discriminant validity, IDV).

The internal consistency reliability of the RTQ was measured using Cronbach’s alpha coefficients [36], which were computed to estimate the internal consistency reliability of each dimension score. A reliability of at least 0.70 is recommended to compare groups of patients [37].

The uni-dimensionality of the new version (RTQ V2.0) was assessed using Rasch analyses, the Partial Credit Model [38,39]. The scalability of each dimension’s scale was assessed by the pattern of the item’s goodness-of-fit statistics (INFIT). Items with INFIT included in the]0.7;1.3 [interval were kept ensuring that all the items of the scale tended to measure the same concept.

External validity and convergent validity were explored by calculating Pearson’s correlation coefficients between the RTQ scores and SF-36 scores. Dimensions measuring the same concept were expected to be correlated with each other.

Finally, we tested the scores of HRQOL of the RTQ V2.0 with comparisons of subgroups (sex and age class) to confirm good properties of measurement compared with results of other studies.

For the third phase, the construct validity was confirmed with a Confirmatory Factor Analysis (CFA) for the second sample of 1,591 patients. Robust maximum likelihood Confirmatory Factor Analysis was performed to test the fit to the model assessed by the computation of the root mean square error of approximation (RMSEA: <0.05, good fit; 0.05–0.08, fair or reasonable; >0.08 unsatisfactory fit), the comparative fit index (CFI), the Standardized Root Mean Square Residual (SRMR) and the Goodness of Fit Index (GFI) [34,40]. The CFI and the GFI are expected to be greater than 0.9 if the fit is adequate and the SRMR must be close to 0 [41-43]. All correlations between each dimension and its items were extracted from the CFA.

Data analyses were performed using SPSS 19.0, Winstep and LISREL softwares.

## Results

The study sample for the first two phases included 1,059 patients; 1,591 patients were enrolled in the third phase. The data set was jointly normally distributed.

The characteristics of the two samples (Table 1) and the HRQOL scores (Table 2) were not significantly different, except concerning the second sample where the proportion of retired patients was slightly higher and the “treatment dimension” which was slightly lower.

**Table 1:** Socio-demographic comparison between the two samples used

		**Population 1 N=1059**	**Population 2 N=1591**	Significance*
Male		61.9% (n=656)	60.5% (n=963)	p=0.52
Age (years), mean ± standard deviation		55.2 ± 12.4	55.3 ± 14.2	p=0.85
Level of education	Secondary 1st stage (college & high school)	45.6% (n=483)	45.3% (n=721)	p=0.9
Employment status	Employed	35.5% (n=377)	39.9% (n=635)	p=0.007
	Retired	29.9% (n=317)	36.1% (n=574)	P<0.001

* Chi-square test for frequency comparison. Student *t*-test for mean comparisons.

**Table 2:** Comparison of RTQ V1 scores between the two samples

	**Population 1**		**Population 2**		***T*-test**
**Dimensions**	**n**	**mean ± SD (min-max)**	**n**	**mean ± SD (min-max)**	**p-value**
**PH**	1039	59.5 ± 18.7 (0–100)	1573	58.8 ± 19.0 (0–100)	**0.34**
**MH**	1042	77.1 ± 19.3 (0–100)	1571	76.5 ± 19.0 (4.8-100)	**0.45**
**MC**	1027	72.7 ± 17.6 (0–100)	1572	72.3 ± 17.8 (0–100)	**0.61**
**TR**	1030	79.0 ± 15.9 (25–100)	1575	75.8 ± 17.8 (8.3-100)	**<0.001**
**FLG**	1042	58.4 ± 20.4 (0–100)	1566	59.8 ± 19.5 (0–100)	**0.07**

*SD*, standard deviation; *PH*, Physical health; *MH*, Mental health; *MC*, Medical care and satisfaction; *TR*, Treatment; *FG*, Fear of losing graft.

For the first phase, the PCA of the RTQ V1 showed an unsatisfactory structure of the items with the dimensions, with a KMO of 0.841. The 5 dimensions were confirmed with a variance of about 50%, but several items were not classified in the appropriate component and had a low psychometric performance (<0.40). At the end of this step, 10 items were removed (Table 3).

**Table 3:** Number of items deleted according to dimensions and versions of RTQ

	**RTQ V1**	**RTQ V2**	**Items deleted**
Dimensions	PH = 10	PH = 8	2
	MH = 9	SF = 5	4
	MC = 11	MC = 8	3
	TR = 9	TR = 5	4
	FLG = 6	FLG = 6	0
Total	**45**	**32**	**13**

*PH*, Physical health; *MH*, Mental health; *MC*, Medical care and satisfaction; *SF*, Social functioning; *TR*, Treatment; *FG*, Fear of losing graft.

The analysis of the response rate, the inter-item correlation and the floor and ceiling effects allowed us to remove 3 more items for which the response rate was <30%, inter-item correlation was 0.845, and a ceiling effect of >70%. Item reduction was performed at the end of the first step, where a total of 13 items were removed (Table 3). The iterative process of item selection resulted in a final version of the RTQ V2.0 comprised of 32 items describing 5 domains: Physical Health, Social Functioning, Medical Care, Treatment and Fear of Losing Graft (Table 4). The set of the 32 items kept and the 13 items deleted was discussed by a pluridisciplinary group (nephrologists, interviewers, and methodologists), who accepted and validated the modifications.

**Table 4:** Principal component analysis of the RTQ V2 with Varimax rotation (n=1059)

	**Component**					**Total variance**
	**1**	**2**	**3**	**4**	**5**	
% of variance	23.8	11.7	6.7	6.1	4.8	53.1
**Item number**						**Dimension Labels**
q1	.645					Physical Health
q3	.682					
q4	.567					
q5	.720					
q6	.602		.303			
q7	.618					
q8	.663					
q9	.708					
q40		.770				Medical Care
q41		.670				
q42		.819				
q43		.849				
q44		.730				
q45		.672				
q46		.729				
q49		.761				
q15			.525			Fear of losing graft
q16			.615			
q29			.642			
q30			.694			
q31			.759			
q32			.740			
q19				.833		Social Functioning (previously Mental Health)
q20				.737		
q21				.596		
q22				.595		
q28				.604		
q11		.363			.544	Treatment
q17					.336	
q37					.758	
q38			.301		.682	
q39					.750	

For the 2nd phase, the Kaiser-Meyer-Olkin Measure of Sampling Adequacy (KMO = 0.880) and Bartlett’s Test of Sphericity (p<0.01) were also used to assess the suitability of the respondent data for factor analysis. The structure of the 5 factors was fixed and supported by PCA with Varimax rotation and accounted for 53.1% of the total variance (Table 4). The inter-dimension correlations were all significant (p<0.001) and ranged from r=0.16 for MC with FLG dimension, to r=0.43 for PH with TR dimension. All other inter-dimension correlations were between r=0.27 and r=0.38.

The correlation between each item and its contributive dimension was higher than the correlation with other dimensions. Internal consistency was acceptable for each dimension (>0.4). Item-discriminant validity (IDV) results were acceptable for every dimension of the RTQ (IDV < IIC ranges). The floor effect ranged from 0% to 0.7%, and the ceiling effect ranged from 0.6% to 11.7%. Cronbach’s alpha was >0.7 in every dimension (0.7 to 0.9), satisfying the internal consistency reliability.

Finally, the overall scalability of the RTQ was satisfactory; all items showed a good fit to the Rasch model within each dimension, and showed INFIT statistics inside the acceptable range (0.7 – 1.3). The dimension scales’ characteristics are summarized in Table 5.

**Table 5:** Dimensions characteristics of the 32-item 2nd version of the ReTransQol (n=1059)

**RTQ dimension (number of items)**	**Mean ± SD**	**IIC**	**IDV**	**MV (%)**	**Floor (%)**	**Ceiling (%)**	**Cronbach’s α**	**INFIT**
**PH (8)**	59.5 ± 18.7	0.61 - 0.75	0.09 - 0.44	1.9	0.1	0.6	0.83	0.81 – 1.3
**SF (5)**	77 ± 19.3	0.65 - 0.77	0.11 - 0.35	1.6	0.3	11.7	0.74	0.83 – 1.22
**MC (8)**	72.7 ± 17.6	0.71 - 0.84	0.09 - 0.33	3	0.3	6.9	0.9	0.74 – 1.3
**TR (5)**	79 ± 15.8	0.42 - 0.76	0.10 - 0.40	2.7	0.7	0.6	0.7	0.88 – 1.29
**FLG (6)**	58.4 ± 20.4	0.60 - 0.74	0.01 - 0.40	1.6	0	9.9	0.78	0.89 – 1.2

*PH*, Physical health; *SF*, Social functioning; *MC*, Medical care and satisfaction; *TR*, Treatment; *FG*, Fear of losing graft; *IIC*, item-internal consistency (min-max of Pearson’s r correlation coefficient); *IDV*, item-discriminant validity (min-max of Pearson’s r correlation coefficient); *MV*, percentage of missing values; *Floor*, floor effect (%); *Ceiling*, ceiling effect (%); *Cronbach’s α*, Cronbach’s alpha consistency coefficient; *INFIT*, min-max Rasch statistics.

The results of the convergent validity of the RTQ V2.0 with SF36 dimensions are shown in Table 6. The PH dimension of the RTQ showed high correlations with Vitality, General Health, Physical Functioning and Bodily Pain (r >0.6). The new SF dimension (previously MH) of the RTQ V2.0 was correlated with Mental Health and Social Functioning.

**Table 6:** Correlation between SF36 and RTQ V2 scores

	**PH**	**SF**	**MC**	**TR**	**FLG**
Physical functioning	**.682**	.227	.141	.217	.229
Role limitations due to physical health	.595	.237	.098	.269	.192
Bodily pain	**.668**	.229	.149	.326	.236
Mental health	.560	**.516**	.297	.394	**.430**
Role limitations due to emotional problems	.494	.328	.127	.263	.231
Social functioning	.556	**.465**	.251	.398	.340
Vitality	**.781**	.340	.306	**.402**	.339
General health	**.704**	.304	.289	**.470**	**.462**

*PH*, Physical health, *SF*, Social functioning; *MC*, Medical care and satisfaction; *TR*, Treatment; *FG*, Fear of losing graft; Data are pearson’s r correlation coefficient; significance of correlation coefficient tests were p<0.01 for all correlations. Bold characters indicate main correlation between RTQ and SF36 dimension.

However, the specific MC dimension was globally uncorrelated or was weakly correlated with the SF36 dimensions (r <0.3). The highest correlations of the TR dimension of the RTQ were with Vitality and General Health. Finally, the FLG dimension was best correlated with General Health and Mental Health.

Comparisons of HRQOL scores of different demographic subgroups (Table 7) showed that women have lower scores in comparison with men in every dimension (p<0.05). Three dimensions differed according to age: PH (p=0.002), MC (p=0.009) and FLG (p=0.019). The PH dimension score is lower between patients >65 years and 35–50 years (post hoc Bonferoni test; p=0.003). In contrast, the MC and the FLG dimensions were lower between patients <35 years than >65 years (post hoc Bonferoni test; p=0.04 and p=0.01).

**Table 7:** HRQOL scores of the RTQ V2 for renal transplant recipients according to gender and age class

			**PH**	**MH**	**MC**	**TR**	**FLG**
**Gender**	**Men**	n	643	645	633	635	643
		Mean ± SD	62 ± 17.6	79.2 ± 17.5	74.1 ± 17	79.8 ± 16	60.3 ± 19.7
	**Women**	n	396	397	394	395	399
		Mean ± SD	55.4 ± 19.7	73.6 ± 21.5	70.4 ± 18.3	77.8 ± 15.5	55.2 ± 21
	*t*-test	p-value	p<0.001	p<0.001	p<0.001	p=0.04	p<0.001
**Age**	**Less than 35 years**	n	67	67	65	65	66
		Mean ± SD	62.6 ± 16.8	81.2 ± 15.3	68.1 ± 18.1	75.8 ± 15.7	51.9 ± 22.3
	**35 to 50 years**	n	267	270	269	269	269
		Mean ± SD	62.6 ± 17	75.9 ± 20.4	70.7 ± 17.6	77.9 ± 15.9	58.2 ± 19.4
	**50 to 65 years**	n	473	475	468	469	475
		Mean ± SD	58.7 ± 19.2	76.3 ± 19.6	73.4 ± 18	79.3 ± 16	58.2 ± 20.6
	**More than 65 years**	n	232	230	225	227	232
		Mean ± SD	56.7 ± 19.5	78.8 ± 18.3	74.7 ± 16.3	80.7 ± 15.5	60.8 ± 20
	ANOVA	p-value	0.002	0.083	0.009	0.082	0.019

*PH*, Physical health; *SF*, Social functioning; *MC*, Medical care and satisfaction; *TR*, Treatment; *FLG*, Fear of losing graft; *ANOVA*, Analysis of variance to compare; *RTQ*, scores between age class.

**Figure 2:**
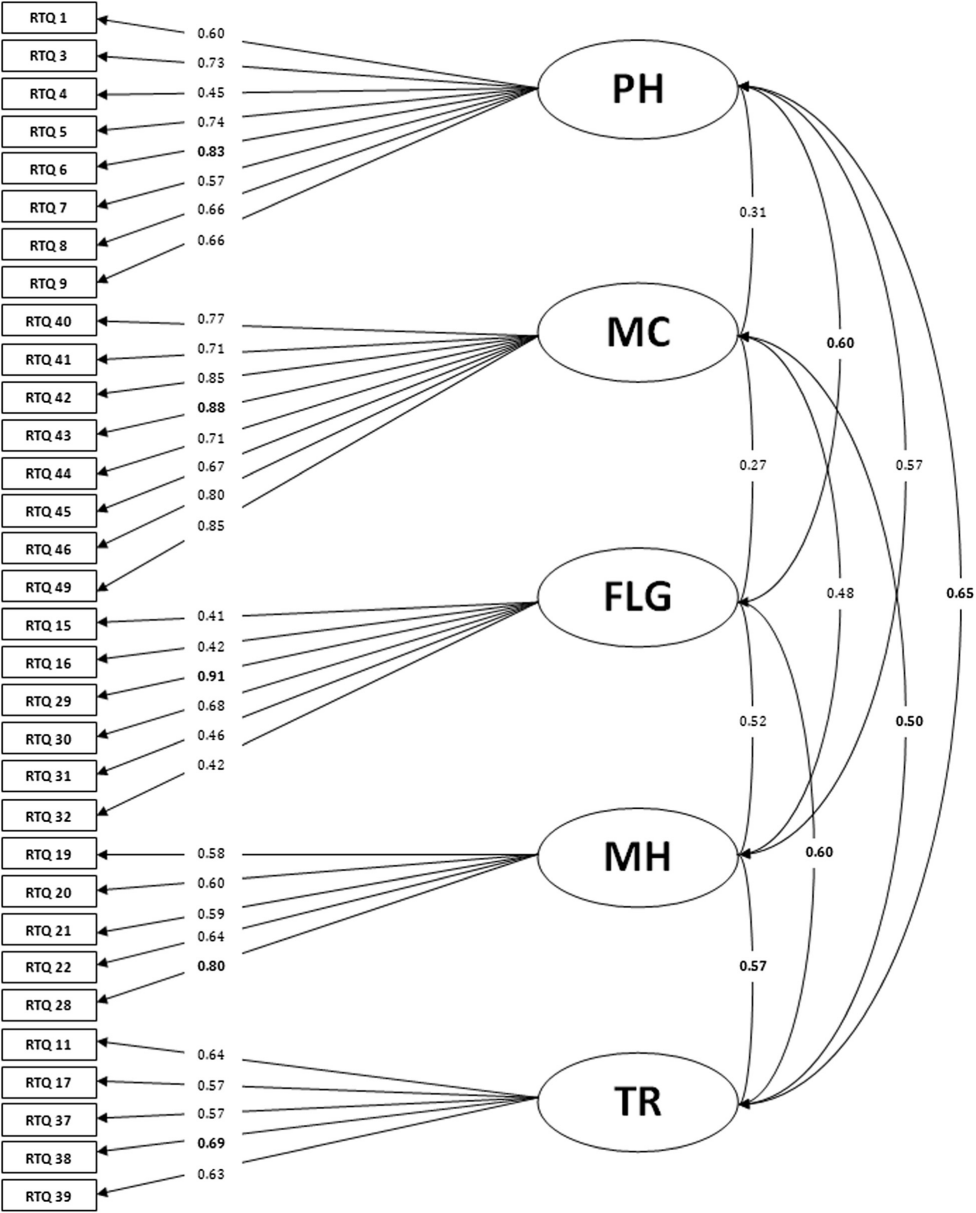
Confirmatory Factor Analysis of the 2nd version of the RTQ.

In the third and last phase, the structure of the RTQ V2.0 was established with a confirmatory factor analysis providing satisfactory indicators: RMSEA = 0.05, SRMR = 0.049, CFI = 0.97 and GFI = 0.91. Strongest correlations of each item in their own dimension and between dimensions are shown in Figure 2 and highlighted in bold.

## Discussion

This paper describes the analysis, item reduction, and validation of a specific self-administered instrument assessing the HRQOL of renal transplant recipients: The ReTransQol (RTQ).

First, we analyzed the structure of the RTQ V1 over a sample of 1,059 RTR from a French cross-sectional multicenter study carried out between March 2007 and March 2008. Our sample size was much larger than those used in other studies to develop HRQOL’s questionnaires, and larger than the first validation of the RTQ (1,059 vs. 355 patients). The construct validity of the RTQ V1 was supported by principal component analysis and showed an unsatisfactory structure, with some correlations below 0.3. However, five dimensions were confirmed by the results of the PCA. At the end of the first phase of the study, 13 items were removed (Table 3) to obtain a better version of the RTQ: the RTQ V2.0. Some items from the first version were moved to other more appropriate dimensions after considering the results of the ACP. The meaning of questions was not changed or modified at all in the second version of the RTQ. Each dimension was named identically to the first version of RTQ, according to the composition of its items, except for Mental Health (MH) which was named Social Functioning (SF) because these items are essentially oriented around the patient’s family, friends and social isolation.

The RTQ V2 is currently comprised of 32 items to specifically assess the HRQOL for the renal transplant recipient. Then, another PCA was performed, which showed a very good structure of the new version of the questionnaire. All items had a factor loading over the recommended threshold of 0.40 for their specific dimension, except for one item (q17: Are you satisfied with your graft?). Nevertheless, item q17 was retained due to the clinical relevance in terms of content validity and to assure a Cronbach’s Alpha ≥0.7 in its dimension. This structure provided better results for reliability, content validity and clinical validity. The explained variance between the first and second RTQ versions improved from 46.3% to 53.1% (an increase of 6.8%).

All psychometric properties of RTQ V2 were satisfactory: IIC >0.4, IDV (%) of 100% and Cronbach’s Alpha >0.7 in every dimension. The acceptability was excellent with a low percentage of missing data and low floor and ceiling effects. The high psychometric properties could be related to the development of the RTQ V2 based on the first version of the RTQ. The new RTQ is shorter than the previous version and should be better accepted by patients, with a shorter average completion time (± −4 min to −5 min).

Convergent validity of the RTQ scores was overall well correlated with that of the generic SF36 questionnaire.

Dimensions relative to physical aspects taken from the SF36 were correctly correlated with the ReTransQol Physical Health dimension, and high correlations were also found between both the Social Functioning dimension and the Mental Health score and ReTransQol Mental Health dimension [renamed Social Functioning (SF)]. Indeed, due to the item reduction step, the 5 items of the previous MH dimension of the RTQ now essentially describe a social functioning dimension.

As expected, the Medical Care dimension was uncorrelated with SF36 dimensions because it is a unique concept of our questionnaire, highlighting the need for a specific HRQOL questionnaire. In addition, strong correlations were found between the Vitality and General Health dimensions of the SF36 and with the Treatment dimension of the RTQ.

Furthermore, as we could expect, the Fear of Losing Graft dimension was correlated with the Mental Health and General Health dimensions of the SF36. All items showed a good fit to the Rasch model within each dimension, and strengthened the unidimensionality of the questionnaire.

Comparisons between different demographic subgroups confirm previous empirical data showing their variations. For example, our results confirm that women have lower QOL scores in comparison with men, in every dimension [44,45]. Concerning age, patients >65 years have a significantly lower score in the PH dimension, with relatively similar scores on the MH and TR dimensions when compared to patients aged 35–50 years, but higher scores on the MC and FLG dimensions compared with younger patients (<35 years). This may be explained by the fact that elderly patients are satisfied with medical care they receive in the hospital, less anguished to lose their graft because of their advanced age, and that they are limited due to their reduced physical capacity [46-48].

Finally, the new version of the RTQ V2 was established by a confirmatory factor analysis with a population of 1,591 RTR. Results of the CFA showed that the model converged with a good index of overall fit (GFI >0.91) and confirmed the satisfactory structure of the new version of the RTQ. Moreover, the CFA showed an interesting correlation between different dimensions: PH and FLG, MC and TR, FLG and PH, FLG and TR, MH and TR, and TR and PH.

Sensitivity to change and reproducibility testing were not performed because the two samples were extracted from two cross-sectional studies. An analysis is currently underway on a French cohort of 334 patients followed over a period of one year.

Although further studies may be needed to test the questionnaire in various cultural contexts, the RTQ V2 (Table 8) should provide a valid measure to assess the HRQOL of RTR.

**Table 8:** Final version of the RTQ V2

**Items number**	**Dimension affectations**	**Items label**	**Response modalities**
1	FLG	Are you anxious about your state of health?	
2	PH	Have you had physical pain?	
3	SF	Do you feel isolated?	All most time
4	PH	Have you felt tired?	Most of time
5	FLG	Do you think about a possible return to dialysis?	A good bit of the time
6	PH	Do you feel energetic?	Some of the time
7	FLG	Does waiting for the results of medical tests distress you or make you feel scared?	A little of the time
8	PH	Do you engage in physical exercise?	None of time
9	FLG	Do you still sometimes think about dialysis?	
10	MC	Do you feel like you are sufficiently informed about the side effects of your treatments?	
11	TR	Are you annoyed by the side effects of treatment?	
12	MC	Are you satisfied by your medical follow-up?	
13	FLG	Do you often think about your graft?	
14	TR	Are you satisfied with your graft?	
15	SF	Does your family offer you moral support?	
16	MC	Do you have trust in the prescribed treatments?	
17	TR	Are you scared of the possible side effects of the anti-rejection treatment?	
18	SF	Do you feel close to your friends?	Not at all
19	TR	Is taking medications a constraint for you?	A little bit
20	MC	Do you feel supported by the medical team?	Moderately
21	TR	Are your doctor’s orders restrictive?	Quite a bit
22	MC	Do you trust your nephrologist?	Extremely
23	SF	Do you feel misunderstood by the people around you?	
24	MC	Do you feel sufficiently informed by your nephrologist?	
25	SF	Has your family accepted your illness?	
26	MC	Do you feel like you are sufficiently informed about complications of the graft?	
27	FLG	Have you been able to forget that you received a graft?	
28	MC	Are you satisfied by your nephrologist’s ability to listen?	
29	PH	You are as well as anyone else	Definitely agree
30	PH	You have stopped doing certain things.	Mostly agree
31	PH	You feel autonomous.	Not agree not disagree
32	PH	You can do your housework and errands by yourself.	Mostly disagree
			Definitely disagree

## Conclusion

This study reported the different phases of analysis, adaptation and validation of the RTQ and the resulting new version: the RTQ V2 (Table 8). It is a HRQOL questionnaire for renal transplant recipients which was developed and improved in response to a lack of any validated instrument for these patients in the French language. The quality of life for patients with chronic diseases has become a public health priority in France, especially since the new Public Health Law.

Psychometric properties allow this new version of the questionnaire to be used to assess different specific dimensions for the renal transplant population, more effectively than previously possible. We envision continuing this work by the follow-up of a prospective cohort to study the sensitivity to change, reproducibility and clinically significant threshold. Furthermore, we plan to adapt and validate this new version of the questionnaire in English.

## Competing interests

The authors declare that they have no competing interests.

## Authors’ contributions

DB performed statistical analysis, analyzed and interpreted the data, collected some data and drafted the manuscript. SG conceived the study and its design, coordinated the data management, revised the manuscript critically; EJ participated in the design of the study, collected the data and performed the statistical analysis BD participated in the design of the study, collected medical data and participated to the interpretation of data. CJ et SB conceived the study and its design, coordinated the data management,revised the manuscript critically for important intellectual content and have given final approval of the version to be published. All authors read and approved the final manuscript.

## References

[B1] Molnar-Varga M Molnar MZ Szeifert L Kovacs AZ Kelemen A Becze A Laszlo G Szentkiralyi A Czira ME Mucsi I Novak M Health-related quality of life and clinical outcomes in kidney transplant recipients Am J Kidney Dis 2011 58 444 452 10.1053/j.ajkd.2011.03.028 21658828

[B2] Nilsson M Forsberg A Bäckman L Lennerling A Persson L-O The perceived threat of the risk for graft rejection and health-related quality of life among organ transplant recipients J Clin Nurs 2011 20 274 282 10.1111/j.1365-2702.2010.03388.x 20964748

[B3] Avramovic M Stefanovic V Health-related quality of life in different stages of renal failure Artif Organs 2012 36 581 589 10.1111/j.1525-1594.2011.01429.x 22428704

[B4] Maglakelidze N Pantsulaia T Tchokhonelidze I Managadze L Chkhotua A Assessment of health-related quality of life in renal transplant recipients and dialysis patients Transplant Proc 2011 43 376 379 10.1016/j.transproceed.2010.12.015 21335226

[B5] Jofré R López-Gómez JM Moreno F Sanz-Guajardo D Valderrábano F Changes in quality of life after renal transplantation Am J Kidney Dis 1998 32 93 100 10.1053/ajkd.1998.v32.pm9669429 9669429

[B6] Chanliau J Kessler M La dialyse péritonéale dans le parcours de soins de l’insuffisant rénal : aspects financiers Néphrol Thérapeutique 2011 7 32 37 21112269 10.1016/j.nephro.2010.10.004

[B7] De Abreu MM Walker DR Sesso RC Ferraz MB A cost evaluation of peritoneal dialysis and hemodialysis in the treatment of end-stage renal disease in Sao Paulo, Brazil Perit Dial Int J Int Soc Perit Dialjuin 2013 33 3 304 315 10.3747/pdi.2011.00138 PMC3649900 23209041

[B8] Suja A Anju R Anju V Neethu J Peeyush P Saraswathy R Economic evaluation of end stage renal disease patients undergoing hemodialysis J Pharm Bioallied Sci 2012 4 107 111 10.4103/0975-7406.94810 22557920 PMC3341713

[B9] Blotière P-O Tuppin P Weill A Ricordeau P Allemand H Coût de la prise en charge de l’IRCT en France en 2007 et impact potentiel d’une augmentation du recours à la dialyse péritonéale et à la greffe Néphrol Thérapeutique 2010 6 240 247 20554257 10.1016/j.nephro.2010.04.005

[B10] Fresenius Medical Care ESRD Patients in 2011 A Global Perspective 2012 http://www.vision-fmc.com/files/download/ESRD/ESRD_Patients_in_2011.pdf

[B11] REIN: rapport annuel 2010 http://www.agence-biomedecine.fr/IMG/pdf/2012_rapport_annuel_rein.pdf

[B12] Djamali A Samaniego M Muth B Muehrer R Hofmann RM Pirsch J Howard A Mourad G Becker BN Medical care of kidney transplant recipients after the first posttransplant year Clin J Am Soc Nephrol 2006 1 623 640 10.2215/CJN.01371005 17699268

[B13] Pauly RP Survival comparison between intensive hemodialysis and transplantation in the context of the existing literature surrounding nocturnal and short-daily hemodialysis Nephrol Dial Transplant 2013 28 44 47 10.1093/ndt/gfs419 23300280

[B14] Wolfe RA Ashby VB Milford EL Ojo AO Ettenger RE Agodoa LY Held PJ Port FK Comparison of mortality in all patients on dialysis, patients on dialysis awaiting transplantation, and recipients of a first cadaveric transplant N Engl J Med 1999 341 1725 1730 10.1056/NEJM199912023412303 10580071

[B15] Abecassis M Bartlett ST Collins AJ Davis CL Delmonico FL Friedewald JJ Hays R Howard A Jones E Leichtman AB Merion RM Metzger RA Pradel F Schweitzer EJ Velez RL Gaston RS Kidney transplantation as primary therapy for End-stage renal disease: a national kidney foundation/kidney disease outcomes quality initiative (NKF/KDOQI^TM^) conference CJASN 2008 3 471 480 18256371 10.2215/CJN.05021107 PMC2390948

[B16] Muehrer RJ Becker BN Life after transplantation: new transitions in quality of life and psychological distress Semin Dial 2005 18 124 131 15771656 10.1111/j.1525-139X.2005.18214.x

[B17] Muehrer RJ Becker BN PSYCHOSOCIAL FACTORS IN PATIENTS WITH CHRONIC KIDNEY DISEASE: life after transplantation: New transitions in quality of life and psychological distress Sem Dialysis 2005 18 124 131 10.1111/j.1525-139X.2005.18214.x 15771656

[B18] Rebollo P González MP Bobes J Saiz P Ortega F [Interpretation of health-related quality of life of patients on replacement therapy in end-stage renal disease] Nefrologia 2000 20 431 439 11100664

[B19] Fiebiger W Mitterbauer C Oberbauer R Health-related quality of life outcomes after kidney transplantation Health Qual Life Outcomes 2004 2 2 10.1186/1477-7525-2-2 14713316 PMC317371

[B20] Lee AJ Morgan CL Conway P Currie CJ Characterisation and comparison of health-related quality of life for patients with renal failure Curr Med Res Opin 2005 21 1777 1783 10.1185/030079905X65277 16307698

[B21] Maglakelidze N Pantsulaia T Managadze L Chkhotua A Assessment of health-related quality of life in living kidney donors Transplant Proc 2011 43 373 375 10.1016/j.transproceed.2010.12.016 21335225

[B22] Gentile S Beauger D Speyer E Jouve E Dussol B Jacquelinet C Factors associated with health-related quality of life in renal transplant recipients: results of a national survey in France Health Qual Life Outcomes 30 mai 2013 11 1 88 23721430 10.1186/1477-7525-11-88 PMC3673846

[B23] Gentile S Delarozière JC Fernandez C Tardieu S Devictor B Dussol B Daurès JP Berland Y Sambuc R Review of quality of life instruments used in end-stage renal disease Nephrologie 2003 24 293 301 14584296

[B24] Parfrey PS Vavasour H Bullock M Henry S Harnett JD Gault MH Development of a health questionnaire specific for end-stage renal disease Nephron 1989 52 20 28 10.1159/000185577 2651947

[B25] Fujisawa M Ichikawa Y Yoshiya K Isotani S Higuchi A Nagano S Arakawa S Hamami G Matsumoto O Kamidono S Assessment of health-related quality of life in renal transplant and hemodialysis patients using the SF-36 health survey Urology 2000 56 201 206 10.1016/S0090-4295(00)00623-3 10925078

[B26] Wight JP Edwards L Brazier J Walters S Payne JN Brown CB The SF36 as an outcome measure of services for end stage renal failure Qual Health Care 1998 7 209 221 10.1136/qshc.7.4.209 10339023 PMC2483621

[B27] Telles-Correia D Barbosa A Mega I [Quality of life and transplantation] Acta Med Port 2010 23 1091 1100 21627885

[B28] Gentile S Jouve E Dussol B Moal V Berland Y Sambuc R Development and validation of a French patient-based health-related quality of life instrument in kidney transplant: the ReTransQoL Health Qual Life Outcomes 2008 6 78 10.1186/1477-7525-6-78 18851730 PMC2577632

[B29] Gentile S Boini S Germain L Jacquelinet C Qualité de vie des patients dialysés et transplantés rénaux: résultats de deux enquêtes multirégionales, France Bull Epidemiol Hebd 2010 9–10 92 96

[B30] Agence de Biomédecine Renal Epidemiology and Information Network: 2008 REIN report Nephrol Ther 2010 6 Suppl 2 S25-183 20728860 10.1016/S1769-7255(10)70009-2

[B31] Ware JE Jr Sherbourne CD The MOS 36-item short-form health survey (SF-36). I. Conceptual framework and item selection Med Care 1992 30 473 483 10.1097/00005650-199206000-00002 1593914

[B32] Hair JF Jr Black WC Babin BJ Anderson RE Multivariate Data Analysis: A Global Perspective 2010 Seventh ed Upper Saddle River, New Jersey: Pearson ISBN-13: 978-0-13-515309-3.

[B33] Kaiser HF The varimax criterion for analytic rotation in factor analysis Psychometrika 1958 23 187 200 10.1007/BF02289233

[B34] Campbell DT Fiske DW Convergent and discriminant validation by the multitrait-multimethod matrix Psychol Bull 1959 56 81 105 13634291

[B35] Carey RG Seibert JH A patient survey system to measure quality improvement: questionnaire reliability and validity Med Care 1993 31 834 845 10.1097/00005650-199309000-00008 8366685

[B36] Cronbach LJ Coefficient alpha and the internal structure of tests Psychometrika 1951 16 297 334 10.1007/BF02310555

[B37] Cortina JM What is coefficient alpha? An examination of theory and applications J App Psychol 1993 78 98 104

[B38] Masters GN A rasch model for partial credit scoring Psychometrika 1982 47 149 174 10.1007/BF02296272

[B39] Masters GN The analysis of partial credit scoring App Measure Educ 1988 1 279 297 10.1207/s15324818ame0104_2

[B40] Jöreskog KG A general approach to confirmatory maximum likelihood factor analysis Psychometrika 1969 34 183 202 10.1007/BF02289343

[B41] Hu L Bentler PM Cutoff criteria for fit indexes in covariance structure analysis: Conventional criteria versus new alternatives Struct Equa Model Multidisciplin J 1999 6 1 55 10.1080/10705519909540118

[B42] Thompson B Exploratory and confirmatory factor analysis: understanding concepts and applications 2004 Washington, DC, US: American Psychological Association

[B43] Baumgartner H Homburg C Applications of structural equation modeling in marketing and consumer research: A review Int J Res Marketing 1996 13 139 161 10.1016/0167-8116(95)00038-0

[B44] Lazzaretti CT Carvalho JGR Mulinari RA Rasia JM Kidney transplantation improves the multidimensional quality of life Transplant Proc 2004 36 872 873 10.1016/j.transproceed.2004.03.094 15194298

[B45] Bohlke M Marini SS Rocha M Terhorst L Gomes RH Barcellos FC Irigoyen MCC Sesso R Factors associated with health-related quality of life after successful kidney transplantation: a population-based study Qual Life Res 2009 18 1185 1193 10.1007/s11136-009-9536-5 19757187

[B46] Valderrábano F Jofre R López-Gómez JM Quality of life in end-stage renal disease patients Am J Kidney Dis 2001 38 443 464 10.1053/ajkd.2001.26824 11532675

[B47] Rosenberger J Van Dijk JP Nagyova I Zezula I Geckova AM Roland R Van den Heuvel WJA Groothoff JW Predictors of perceived health status in patients after kidney transplantation Transplantation 2006 81 1306 1310 10.1097/01.tp.0000209596.01164.c9 16699459

[B48] Rebollo P Ortega F Baltar JM Díaz-Corte C Navascués RA Naves M Ureña A Badía X Alvarez-Ude F Alvarez-Grande J Health-related quality of life (HRQOL) in end stage renal disease (ESRD) patients over 65 years Geriatr Nephrol Urol 1998 8 85 94 10.1023/A:1008338802209 9893216

